# Crystal structure and Hirshfeld surface analysis of isopropyl 4-[2-fluoro-5-(tri­fluoro­meth­yl)phen­yl]-2,6,6-trimethyl-5-oxo-1,4,5,6,7,8-hexa­hydro­quinoline-3-carboxyl­ate

**DOI:** 10.1107/S205698902300141X

**Published:** 2023-02-21

**Authors:** Sema Öztürk Yıldırım, Mehmet Akkurt, Gökalp Çetin, Rahime Şimşek, Ray J. Butcher, Ajaya Bhattarai

**Affiliations:** a Department of Physics, Faculty of Science, Eskisehir Technical University, Yunus Emre Campus 26470 Eskisehir, Türkiye; b Department of Physics, Faculty of Science, Erciyes University, 38039 Kayseri, Türkiye; c Department of Pharmaceutical Chemistry, Faculty of Pharmacy, Erzincan Binali Yıldırım University, 24100 Erzincan, Türkiye; d Department of Pharmaceutical Chemistry, Faculty of Pharmacy, Hacettepe University, 06100 Sıhhiye-Ankara, Türkiye; e Department of Chemistry, Howard University, Washington DC 20059, USA; f Department of Chemistry, M.M.A.M.C (Tribhuvan University), Biratnagar, Nepal; University of Neuchâtel, Switzerland

**Keywords:** crystal structure, 1,4-di­hydro­pyridine ring, cyclo­hexene ring, quinoline ring system, van der Waals inter­actions, Hirshfeld surface analysis

## Abstract

In the title compound, C_23_H_25_F_4_NO_3_, the 1,4-di­hydro­pyridine ring adopts a distorted boat conformation, while the cyclo­hexene ring is almost showing a half-chair conformation. In the crystal, inter­molecular N—H⋯O hydrogen bonds connect the mol­ecules into chains with graph-set motif *C*(6) parallel to the *a*-axis. These chains are linked together by C—H⋯O and C—H⋯F inter­actions, forming a three-dimensional network. In addition, C—H⋯π inter­actions link the mol­ecules into layers parallel to the (100) plane. A Hirshfeld surface analysis was performed to further investigate the inter­molecular inter­actions.

## Abstract 2

In the crystal, an infinite chain along the *a*-axis direction with a *C*(6) chain motif is formed by N—H⋯O hydrogen bonds. C—H⋯O and C—H⋯F inter­actions connect these chains, generating a three-dimensional network. In addition, C—H⋯π inter­actions connect the mol­ecules into layers parallel to the (100) plane.

## 1. Chemical context

5-Oxo-1,4,5,6,7,8-hexa­hydro­quinoline (5-oxo-HHQ) is a condensed heterocycle, which is formed with di­hydro­pyridine (DHP) and cyclo­hexa­none. In recent years, compounds containing the 5-oxo-HHQ scaffold have been widely studied because of their diverse pharmacological and biological attributes (Ranjbar *et al.*, 2019 ▸). 
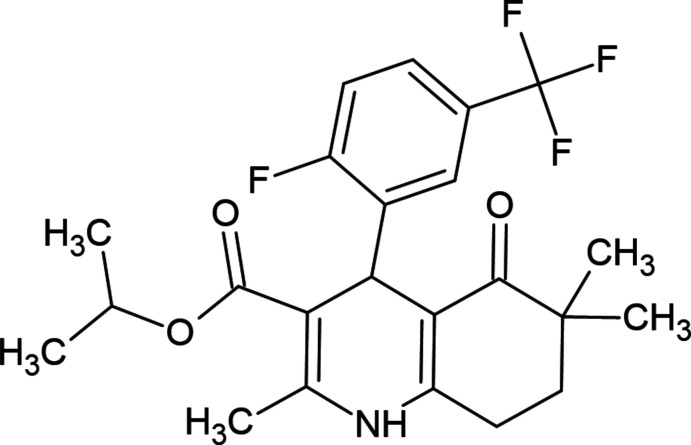


In this study, isopropyl 4-[2-fluoro-5-(tri­fluoro­meth­yl)phen­yl]-2,6,6-trimethyl-5-oxo-1,4,5,6,7,8-hexa­hydro­quinoline-3-carboxyl­ate was synthesized and its mol­ecular structure was confirmed by IR, ^1^H NMR, ^13^C NMR, HRMS and X-ray crystallography. The inter­molecular inter­actions observed in the crystal packing were investigated by Hirshfeld surface analysis.

## 2. Structural commentary

**Figure 1:**
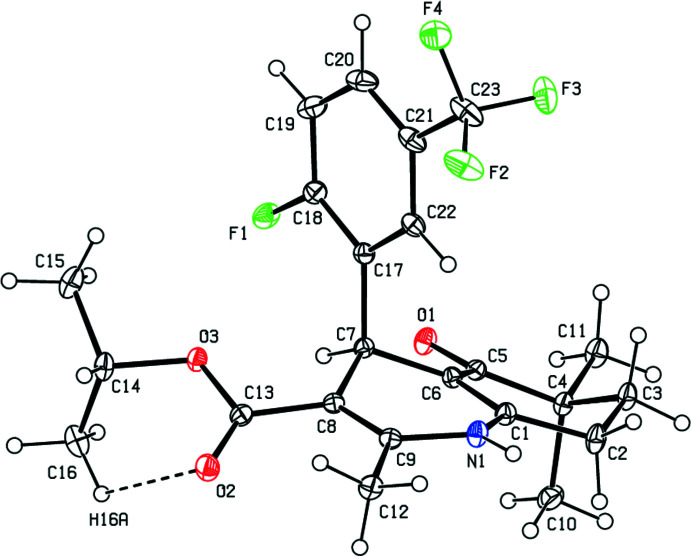
View of the title mol­ecule. Displacement ellipsoids are drawn at the 30% probability level. For clarity, only the major disorder components are included.

As shown in Fig. 1 ▸, the 1,4-di­hydro­pyridine ring (N1/C1/C6-C9) adopts a distorted boat conformation [puckering parameters (Cremer & Pople, 1975 ▸): *Q*_T_ = 0.3164 (16) Å, θ = 75.3 (3)°, φ = 180.0 (3)°], while the cyclo­hexene ring (C1–C6) shows a twisted boat conformation [puckering parameters: *Q*_T_ = 0.4602 (18) Å, θ = 122.0 (2)°, φ = 312.6 (3)°]. The 1-fluoro-4-(tri­fluoro­meth­yl)benzene ring (C17–C22) makes a dihedral angle of 87.91 (8)° with the mean plane of the quinoline ring system [N1/C1–C9; maximum deviation = 0.975 (2) Å for C4]. The geometrical parameter values of the the title compound are in agreement with those reported for similar compounds in the *Database survey* section.

## 3. Supra­molecular features and Hirshfeld surface analysis

**Figure 2:**
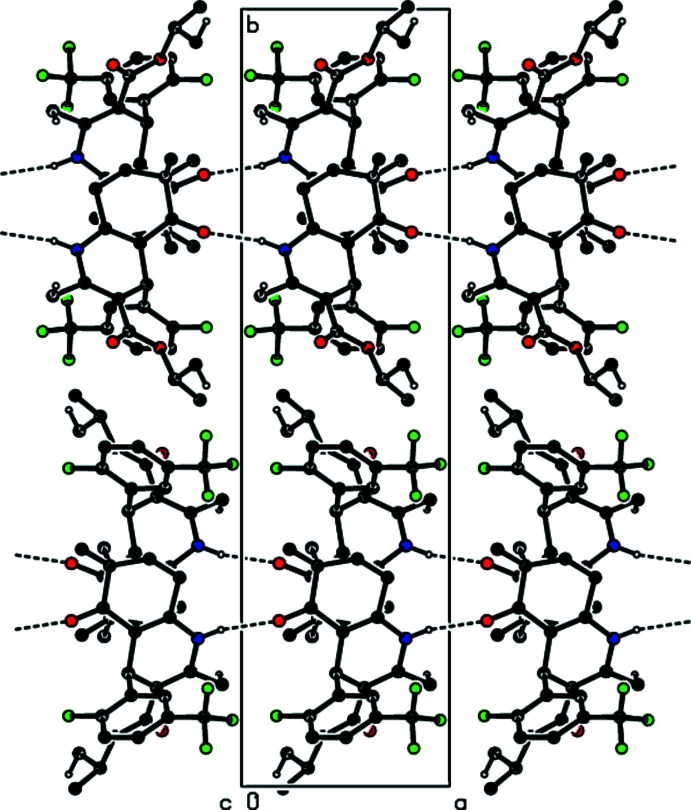
A view of the mol­ecular packing of the title compound, showing the N—H⋯O hydrogen bonds. Only the major components of the disordered atoms are shown.

**Figure 3:**
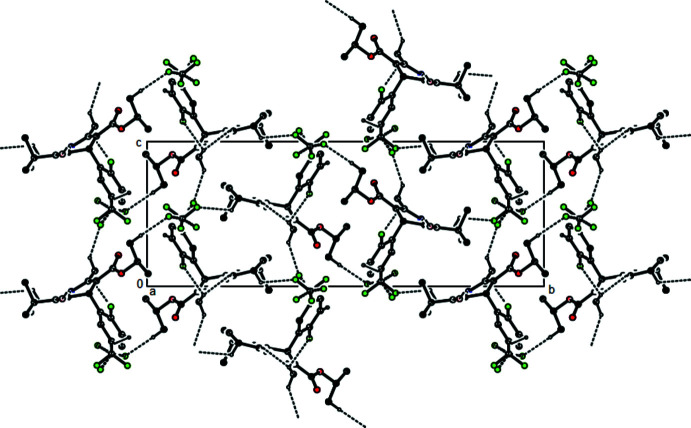
A view of the mol­ecular packing of the title compound, showing the N—H⋯O, C—H⋯O and C—H⋯F hydrogen bonds.

**Figure 4:**
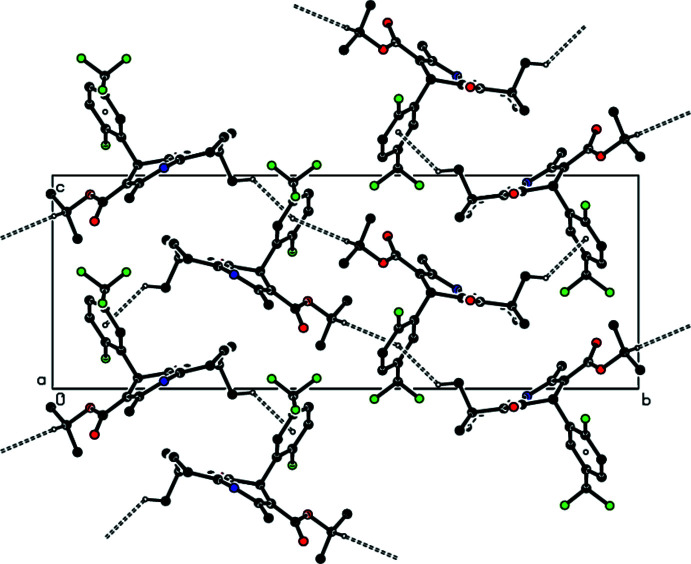
A view of the mol­ecular packing of the title compound, showing the C—H⋯π inter­actions. Only the major components of the disordered atoms are shown.

In the crystal, N—H⋯O hydrogen bonds link the mol­ecules into infinite chains with a a *C*(6) chain motif (Bernstein *et al.*, 1995 ▸) along the *a*-axis direction (Table 1 ▸ and Fig. 2 ▸). These chains are linked together by C—H⋯O and C—H⋯F inter­actions (Table 1 ▸ and Fig. 3 ▸), forming a three-dimensional network. C—H⋯π inter­actions link the mol­ecules into layers parallel to the (100) plane (Table 1 ▸ and Fig. 4 ▸).

**Figure 5:**
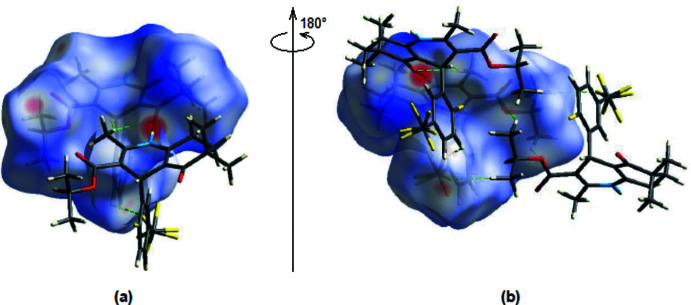
(*a*) Front and (*b*) back views of the three-dimensional Hirshfeld surface for the title compound. Some N—H⋯O, C—H⋯O and C—H⋯F inter­actions are shown as dashed lines.

The Hirshfeld surface analysis of mol­ecular crystal structures is an attempt to go beyond crystal packing diagrams with mol­ecules represented by different patterns and inter­nuclear distances and angles. *Crystal Explorer 17.5* (Turner *et al.*, 2017 ▸) was used to construct Hirshfeld surfaces for the title compound. The *d*_norm_ mappings for the title compound were performed in the range of −0.4718 to +1.7749 a.u. On the *d*_norm_ surfaces, bright red spots show the locations of the N—H⋯O, C—H⋯O and C—H⋯F inter­actions (Tables 1 ▸ and 2 ▸; Fig. 5 ▸ *a,b*).

**Figure 6:**
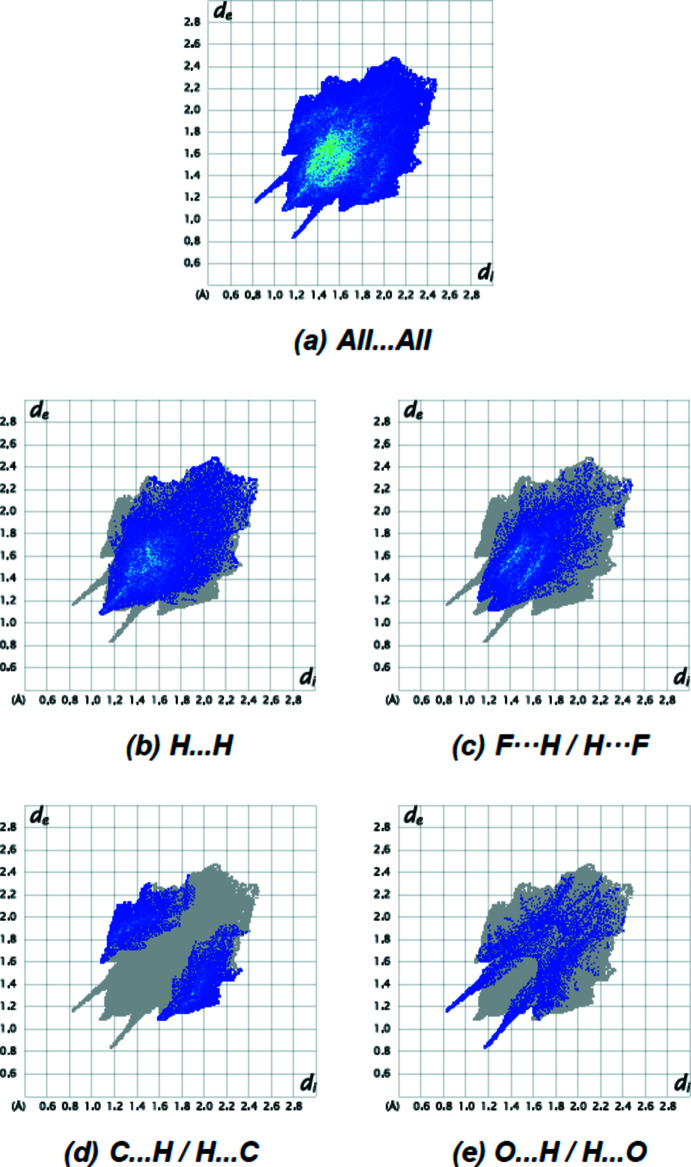
The two-dimensional fingerprint plots for the title compound showing (*a*) all inter­actions, and delineated into (*b*) H⋯H, (*c*) F⋯H/H⋯F, (*d*) C⋯H/H⋯C and (*e*) O⋯H/H⋯O inter­actions. The *d*_i_ and *d*_e_ values are the closest inter­nal and external distances (in Å) from given points on the Hirshfeld surface.

The overall two-dimensional fingerprint plot for the title compound and those delineated into H⋯H (Fig. 6 ▸ *b*; 42.3), F⋯H/H⋯F (Fig. 6 ▸ *c*; 28.5%), C⋯H/H⋯C (Fig. 6 ▸ *d*; 14.6%) and O⋯H/H⋯O (Fig. 6 ▸ *e*; 10.8%) contacts are shown in Fig. 6 ▸. F⋯O/O⋯F (1.8%), F⋯F (1.3%), N⋯H/H⋯N (0.5%) and F⋯C/C⋯F (0.2%) contacts have little directional influence on the mol­ecular packing.

## 4. Database survey

A search of the Cambridge Structural Database (CSD, Version 5.42, update of September 2021; Groom *et al.*, 2016 ▸) for similar structures with the 1,4,5,6,7,8-hexa­hydro­quinoline group showed that the eight most closely related to the title compound are refcodes ECUCUE [(**I**); Yıldırım *et al.*, 2022 ▸], LOQCAX [(**II**); Steiger *et al.*, 2014 ▸), NEQMON [(**III**); Öztürk Yıldırım *et al.*, 2013 ▸], PECPUK [(**IV**); Gündüz *et al.*, 2012 ▸] IMEJOA [(**V**); Linden *et al.*, 2011 ▸], PUGCIE [(**VI**); Mookiah *et al.*, 2009 ▸], UCOLOO [(**VII**); Linden *et al.*, 2006 ▸] and DAYJET [(**VIII**); Linden *et al.*, 2005 ▸]. Mol­ecules of all these compounds are linked by N—H⋯O hydrogen bonds. Additionally, C—H⋯O hydrogen bonds in (**I**), (**III**), (**V**) and (**VI**) and C—H⋯π inter­actions in (**I**) were also observed.

## 5. Synthesis and crystallization

The compound was obtained by a modified one-pot Hantzsch synthesis, which consists of refluxing 4,4-dimethyl-1,3-cyclo­hexa­nedione (1 mmol), isopropyl aceto­acetate (1 mmol) and 2-fluoro-5-(tri­fluoro­meth­yl)benzaldehyde (1 mmol) in methanol in the presence of ammonium acetate (5 mmol). The reaction was monitored by TLC using ethyl acetate–*n*-hexane (1:1). The reaction mixture was cooled down to room temperature and then poured into ice–water. The precipitated solid was filtered and crystallized from methanol (Çetin *et al.*, 2022 ▸).

***Isopropyl 2,6,6-trimethyl-4-(3-fluoro-5-tri­fluoro­methyl­phen­yl)-5-oxo-1,4,5,6,7,8-hexa­hydro­quinoline-3-carboxyl­ate.*** Yellowish solid, m.p: 469–471 K, yield: 60%. IR (cm^−1^) 3299 (N—H), 1697 (C=O, ester), 1646 (C=O, ketone), ^1^H NMR (400 MHz, DMSO-*d*_6_): *δ* 0.82 (3H, *s*, 6-CH_3_), 0.91 [3*H*, *d*, *J =* 6.4 Hz, CH**(CH_3_)_2a_**], 0.97 (3H, *s*, 6-CH_3_), 1.16 [3*H*, d, *J* = 6.4, CH**(CH_3_)_2b_**], 1.64–1.76 (2H, *m*, quinoline H7), 2.26 (3H, *s*, 2-CH_3_), 2.48–2.51 (2H, *m*, quinoline H8), 4.75–4.81 [H, *m*, **CH**(CH_3_)_2_], 5.02 (H, *s*, quinoline H4), 7.24 (H, *dd*, *J* = 9.2, 6.8 Hz, Ar-H3), 7.50–7.54 (2H, *m*, Ar-H), 9.21 (H, *s*, NH). ^13^C NMR (100 MHz, DMSO-*d*_6_): *δ* 18.2 (2-CH_3_), 21.2 [COOCH**(CH_3_)_2a_**], 21.7 [COOCH**(CH_3_)_2b_**], 22.9 (C-8), 24.2 (6-CH_3_), 24.7 (6-CH_3_), 33.1 (C-7), 34.0 (C-4), 39.4 (C-6), 66.0 [COO**CH**(CH_3_)_2_], 101.2 (C-3), 107.5 (C-4a), 116.4, 122.7, 125.4, 128.2, 135.5, 163.4 (phenyl carbons), 125.1 (CF_3_), 146.1 (C-2), 150.4 (C-8a), 165.9 [**C**OOCH(CH_3_)_2_], 199.3 (C-5). HRMS (ESI/Q-TOF) *m*/*z*: [*M* + H]^+^ calculated for C_23_H_25_F_4_NO_3:_ 440.1804; found: 440.1975.

## 6. Refinement

Crystal data, data collection and structure refinement details are summarized in Table 3 ▸. All C-bound H atoms were placed in geometrically idealized positions (C—H = 0.95–1.00 Å) while the hydrogen atom attached to N1 was found in a difference map, and was subsequently refined freely [N1—H1*N* = 0.88 (2) Å]. All C-bound H atoms were included as riding contributions with isotropic displacement parameters 1.2 times those of the parent atoms (1.5 for methyl groups). All F atoms of the tri­fluoro­methyl unit of the mol­ecule are disordered over two sites [relative occupancies 0.763 (5):0.237 (5)]. DFIX, SIMU and DELU instructions were used to restrain the disordered F atoms.

## Supplementary Material

Crystal structure: contains datablock(s) I. DOI: 10.1107/S205698902300141X/tx2062sup1.cif

Structure factors: contains datablock(s) I. DOI: 10.1107/S205698902300141X/tx2062Isup2.hkl

Supporting information file. DOI: 10.1107/S205698902300141X/tx2062Isup3.cml

CCDC reference: 2242647

Additional supporting information:  crystallographic information; 3D view; checkCIF report

## Figures and Tables

**Table 1:** Hydrogen-bond geometry (Å, °) *Cg*3 is the centroid of the C17–C22 ring.

*D*—H⋯*A*	*D*—H	H⋯*A*	*D*⋯*A*	*D*—H⋯*A*
N1—H1*N*⋯O1^i^	0.88 (2)	2.12 (2)	2.9798 (19)	165 (2)
C2—H2*A*⋯F3^ii^	0.99	2.59	3.187 (4)	119
C12—H12*B*⋯F1^i^	0.98	2.64	3.206 (2)	117
C12—H12*B*⋯O1^i^	0.98	2.65	3.505 (2)	145
C12—H12*C*⋯F3*A* ^iii^	0.98	2.43	3.243 (15)	141
C16—H16*A*⋯O2	0.98	2.59	3.106 (2)	113
C16—H16*C*⋯F4*A* ^iv^	0.98	2.43	3.409 (6)	174
C19—H19*A*⋯F2^v^	0.95	2.52	3.117 (3)	121
C10—H10*C*⋯*Cg*3^ii^	0.98	2.93	3.631 (2)	130
C14—H14*A*⋯*Cg*3^iv^	1.00	2.91	3.7707 (18)	145

Symmetry codes: (i) 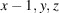 ; (ii) 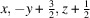 ; (iii) 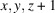 ; (iv) 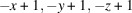 ; (v) 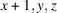 .

**Table 2:** Summary of short inter­atomic contacts (Å)

O1⋯H1*N*	2.12 (2)	1 + *x*, *y*, *z*
F3*A*⋯H12*C*	2.43	*x*, *y*, −1 + *z*
F3⋯H2*A*	2.59	*x*, 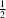 − *y*, − 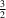 + *z*
F4*A*⋯H16*C*	2.58	−1 + *x*, *y*, −1 + *z*
H16*C*⋯F4*A*	2.43	1 − *x*, 1 − *y*, 1 − *z*
F2*A*⋯H10*A*	2.81	−1 + *x*, 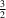 − *y*, − 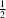 + *z*
H15*C*⋯H15*C*	2.41	2 − *x*, 1 − *y*, 1 − *z*
H20*A*⋯H20*A*	2.52	1 − *x*, 1 − *y*, −*z*

**Table 3:** Experimental details

Crystal data	
Chemical formula	C_23_H_25_F_4_NO_3_
*M*_r_	439.44
Crystal system, space group	Monoclinic, *P*2_1_/*c*
Temperature (K)	100
*a*, *b*, *c* (Å)	7.4918 (3), 27.8140 (11), 10.2023 (4)
β (°)	97.053 (2)
*V* (Å^3^)	2109.84 (14)
*Z*	4
Radiation type	Mo *K*α
μ (mm^−1^)	0.11
Crystal size (mm)	0.23 × 0.17 × 0.10

Data collection	
Diffractometer	Bruker APEXII CCD
Absorption correction	Multi-scan (*SADABS*; Krause *et al.*, 2015 ▸)
*T*_min_, *T*_max_	0.663, 0.746
No. of measured, independent and observed [*I* > 2σ(*I*)] reflections	21749, 5221, 3801
*R*_int_	0.056
(sin θ/λ)_max_ (Å^−1^)	0.667

Refinement	
*R*[*F* ^2^ > 2σ(*F* ^2^)], *wR*(*F* ^2^), *S*	0.046, 0.129, 1.08
No. of reflections	5221
No. of parameters	318
No. of restraints	48
H-atom treatment	H atoms treated by a mixture of independent and constrained refinement
Δρ_max_, Δρ_min_ (e Å^−3^)	0.35, −0.37

Computer programs: *APEX3* and *SAINT* (Bruker, 2018 ▸), *SHELXT* (Sheldrick, 2015*a* ▸), *SHELXL* (Sheldrick, 2015*b* ▸), *ORTEP-3 for Windows* (Farrugia, 2012 ▸) and *PLATON* (Spek, 2020 ▸).

## supplementary crystallographic information

### Crystal data

**Table:**

C_23_H_25_F_4_NO_3_	*F*(000) = 920
*M**_r_* = 439.44	*D*_x_ = 1.383 Mg m^−^^3^
Monoclinic, *P*2_1_/*c*	Mo *K*α radiation, λ = 0.71073 Å
*a* = 7.4918 (3) Å	Cell parameters from 8405 reflections
*b* = 27.8140 (11) Å	θ = 2.5–28.2°
*c* = 10.2023 (4) Å	µ = 0.11 mm^−^^1^
β = 97.053 (2)°	*T* = 100 K
*V* = 2109.84 (14) Å^3^	Prism, yellowish
*Z* = 4	0.23 × 0.17 × 0.10 mm

### Data collection

**Table:**

Bruker APEXII CCD diffractometer	3801 reflections with *I* > 2σ(*I*)
φ and ω scans	*R*_int_ = 0.056
Absorption correction: multi-scan (SADABS; Krause *et al.*, 2015)	θ_max_ = 28.3°, θ_min_ = 2.5°
*T*_min_ = 0.663, *T*_max_ = 0.746	*h* = −9→9
21749 measured reflections	*k* = −36→36
5221 independent reflections	*l* = −13→13

### Refinement

**Table:**

Refinement on *F*^2^	48 restraints
Least-squares matrix: full	Hydrogen site location: mixed
*R*[*F*^2^ > 2σ(*F*^2^)] = 0.046	H atoms treated by a mixture of independent and constrained refinement
*wR*(*F*^2^) = 0.129	*w* = 1/[σ^2^(*F*_o_^2^) + (0.044*P*)^2^ + 1.2617*P*] where *P* = (*F*_o_^2^ + 2*F*_c_^2^)/3
*S* = 1.08	(Δ/σ)_max_ = 0.001
5221 reflections	Δρ_max_ = 0.35 e Å^−^^3^
318 parameters	Δρ_min_ = −0.37 e Å^−^^3^

### Special details

**Table:**

Geometry. All esds (except the esd in the dihedral angle between two l.s. planes) are estimated using the full covariance matrix. The cell esds are taken into account individually in the estimation of esds in distances, angles and torsion angles; correlations between esds in cell parameters are only used when they are defined by crystal symmetry. An approximate (isotropic) treatment of cell esds is used for estimating esds involving l.s. planes.

### Fractional atomic coordinates and isotropic or equivalent isotropic displacement parameters (Å^2^)

**Table:**

	*x*	*y*	*z*	*U*_iso_*/*U*_eq_	Occ. (<1)
F1	0.83123 (14)	0.59076 (4)	0.35900 (11)	0.0285 (3)	
F2	0.0433 (2)	0.58544 (9)	0.09930 (16)	0.0425 (7)	0.763 (5)
F3	0.1611 (9)	0.62597 (14)	−0.0465 (4)	0.0398 (9)	0.763 (5)
F4	0.1702 (3)	0.54913 (6)	−0.04875 (18)	0.0370 (5)	0.763 (5)
F2A	0.0323 (7)	0.6241 (3)	0.0914 (6)	0.056 (2)	0.237 (5)
F3A	0.167 (3)	0.6171 (5)	−0.0756 (14)	0.049 (4)	0.237 (5)
F4A	0.0756 (10)	0.5551 (2)	0.0171 (8)	0.059 (3)	0.237 (5)
O1	0.81859 (16)	0.71304 (4)	0.41408 (13)	0.0227 (3)	
O2	0.38263 (18)	0.57179 (5)	0.71526 (13)	0.0288 (3)	
O3	0.60880 (16)	0.56439 (4)	0.58997 (12)	0.0199 (3)	
N1	0.20965 (19)	0.69030 (5)	0.46495 (15)	0.0207 (3)	
H1N	0.099 (3)	0.7016 (8)	0.459 (2)	0.031 (6)*	
C1	0.3454 (2)	0.71772 (6)	0.42567 (16)	0.0190 (3)	
C2	0.2984 (2)	0.76895 (6)	0.3917 (2)	0.0255 (4)	
H2A	0.285175	0.786980	0.473603	0.031*	
H2B	0.181746	0.770062	0.334509	0.031*	
C3	0.4424 (2)	0.79280 (6)	0.32062 (18)	0.0232 (4)	
H3A	0.419816	0.827862	0.315943	0.028*	
H3B	0.432982	0.780416	0.229056	0.028*	
C4	0.6335 (2)	0.78392 (6)	0.38858 (17)	0.0190 (3)	
C5	0.6655 (2)	0.72984 (6)	0.40730 (16)	0.0175 (3)	
C6	0.5124 (2)	0.69908 (5)	0.42489 (15)	0.0170 (3)	
C7	0.5444 (2)	0.64570 (5)	0.45008 (16)	0.0164 (3)	
H7A	0.668292	0.641386	0.497582	0.020*	
C8	0.4092 (2)	0.62727 (5)	0.53860 (16)	0.0170 (3)	
C9	0.2458 (2)	0.64791 (6)	0.53611 (16)	0.0185 (3)	
C10	0.6615 (3)	0.80687 (7)	0.52669 (19)	0.0293 (4)	
H10A	0.783401	0.799820	0.568766	0.044*	
H10B	0.573604	0.793666	0.580624	0.044*	
H10C	0.645383	0.841759	0.518702	0.044*	
C11	0.7685 (2)	0.80522 (6)	0.30387 (19)	0.0261 (4)	
H11A	0.756447	0.789038	0.217908	0.039*	
H11B	0.890814	0.800633	0.348417	0.039*	
H11C	0.744789	0.839655	0.290953	0.039*	
C12	0.0930 (2)	0.63118 (6)	0.60675 (18)	0.0223 (3)	
H12A	0.092211	0.595962	0.609755	0.033*	
H12B	−0.020875	0.642682	0.559688	0.033*	
H12C	0.107879	0.643951	0.696950	0.033*	
C13	0.4600 (2)	0.58570 (6)	0.62451 (17)	0.0197 (3)	
C14	0.6800 (2)	0.52376 (6)	0.67080 (18)	0.0233 (4)	
H14A	0.579391	0.502405	0.690737	0.028*	
C15	0.8005 (3)	0.49721 (7)	0.5873 (2)	0.0320 (4)	
H15A	0.732182	0.488837	0.502190	0.048*	
H15B	0.845165	0.467797	0.632940	0.048*	
H15C	0.902333	0.517761	0.572403	0.048*	
C16	0.7809 (3)	0.54265 (7)	0.79772 (19)	0.0321 (4)	
H16A	0.698202	0.560747	0.846536	0.048*	
H16B	0.878266	0.563824	0.777245	0.048*	
H16C	0.831503	0.515642	0.851701	0.048*	
C17	0.5305 (2)	0.61730 (5)	0.32111 (16)	0.0180 (3)	
C18	0.6711 (2)	0.59070 (6)	0.28216 (17)	0.0221 (3)	
C19	0.6575 (3)	0.56336 (6)	0.16787 (19)	0.0297 (4)	
H19A	0.757188	0.545153	0.146365	0.036*	
C20	0.4966 (3)	0.56302 (7)	0.08575 (18)	0.0310 (4)	
H20A	0.484741	0.545016	0.006024	0.037*	
C21	0.3531 (3)	0.58922 (7)	0.12102 (17)	0.0261 (4)	
C22	0.3689 (2)	0.61593 (6)	0.23693 (17)	0.0216 (3)	
H22A	0.268204	0.633535	0.259244	0.026*	
C23	0.1789 (3)	0.58922 (8)	0.03321 (19)	0.0355 (5)	

### Atomic displacement parameters (Å^2^)

**Table:**

	*U*^11^	*U*^22^	*U*^33^	*U*^12^	*U*^13^	*U*^23^
F1	0.0201 (5)	0.0332 (6)	0.0326 (6)	0.0071 (4)	0.0043 (4)	−0.0038 (5)
F2	0.0219 (8)	0.0783 (19)	0.0270 (8)	−0.0104 (9)	0.0018 (6)	−0.0014 (9)
F3	0.0451 (19)	0.0281 (10)	0.0418 (19)	−0.0055 (10)	−0.0127 (16)	0.0106 (11)
F4	0.0431 (12)	0.0323 (8)	0.0324 (9)	−0.0078 (7)	−0.0088 (8)	−0.0090 (7)
F2A	0.025 (3)	0.098 (6)	0.041 (3)	0.019 (3)	−0.011 (2)	−0.028 (4)
F3A	0.037 (5)	0.073 (8)	0.037 (6)	0.002 (6)	0.012 (5)	0.026 (5)
F4A	0.051 (4)	0.042 (3)	0.073 (6)	−0.023 (3)	−0.033 (4)	0.020 (4)
O1	0.0149 (6)	0.0213 (6)	0.0324 (7)	0.0021 (5)	0.0044 (5)	0.0027 (5)
O2	0.0299 (7)	0.0294 (6)	0.0297 (7)	0.0054 (5)	0.0138 (6)	0.0089 (5)
O3	0.0180 (6)	0.0186 (5)	0.0236 (6)	0.0031 (4)	0.0045 (5)	0.0039 (5)
N1	0.0119 (7)	0.0219 (7)	0.0285 (8)	0.0021 (5)	0.0039 (6)	0.0045 (6)
C1	0.0152 (8)	0.0202 (8)	0.0216 (8)	0.0017 (6)	0.0031 (6)	0.0013 (6)
C2	0.0171 (8)	0.0226 (8)	0.0372 (10)	0.0054 (6)	0.0055 (7)	0.0084 (7)
C3	0.0187 (9)	0.0198 (8)	0.0312 (9)	0.0051 (6)	0.0041 (7)	0.0068 (7)
C4	0.0168 (8)	0.0164 (7)	0.0238 (8)	0.0016 (6)	0.0029 (6)	0.0021 (6)
C5	0.0167 (8)	0.0187 (7)	0.0172 (7)	0.0013 (6)	0.0023 (6)	0.0000 (6)
C6	0.0161 (8)	0.0176 (7)	0.0171 (7)	0.0005 (6)	0.0016 (6)	0.0013 (6)
C7	0.0135 (7)	0.0177 (7)	0.0179 (7)	0.0012 (6)	0.0014 (6)	−0.0006 (6)
C8	0.0166 (8)	0.0172 (7)	0.0172 (7)	−0.0010 (6)	0.0019 (6)	−0.0013 (6)
C9	0.0182 (8)	0.0191 (7)	0.0181 (7)	−0.0018 (6)	0.0015 (6)	−0.0015 (6)
C10	0.0331 (11)	0.0224 (8)	0.0322 (10)	0.0024 (7)	0.0035 (8)	−0.0053 (7)
C11	0.0208 (9)	0.0215 (8)	0.0369 (10)	−0.0006 (7)	0.0071 (8)	0.0058 (7)
C12	0.0167 (8)	0.0245 (8)	0.0266 (9)	−0.0009 (6)	0.0068 (7)	0.0013 (7)
C13	0.0177 (8)	0.0198 (7)	0.0217 (8)	0.0008 (6)	0.0029 (6)	−0.0015 (6)
C14	0.0201 (9)	0.0186 (8)	0.0314 (9)	0.0017 (6)	0.0036 (7)	0.0077 (7)
C15	0.0261 (10)	0.0225 (9)	0.0485 (12)	0.0055 (7)	0.0082 (9)	0.0031 (8)
C16	0.0314 (11)	0.0334 (10)	0.0298 (10)	−0.0021 (8)	−0.0026 (8)	0.0104 (8)
C17	0.0205 (8)	0.0161 (7)	0.0178 (7)	−0.0029 (6)	0.0044 (6)	0.0013 (6)
C18	0.0220 (9)	0.0213 (8)	0.0237 (8)	−0.0006 (6)	0.0055 (7)	0.0002 (6)
C19	0.0375 (11)	0.0234 (9)	0.0311 (10)	−0.0030 (8)	0.0157 (9)	−0.0056 (7)
C20	0.0448 (12)	0.0281 (9)	0.0220 (9)	−0.0142 (8)	0.0109 (8)	−0.0067 (7)
C21	0.0305 (10)	0.0294 (9)	0.0181 (8)	−0.0139 (7)	0.0012 (7)	0.0030 (7)
C22	0.0221 (9)	0.0240 (8)	0.0190 (8)	−0.0048 (7)	0.0034 (7)	0.0032 (6)
C23	0.0409 (12)	0.0448 (11)	0.0196 (9)	−0.0198 (9)	−0.0006 (8)	0.0048 (8)

### Geometric parameters (Å, º)

**Table:**

F1—C18	1.350 (2)	C8—C13	1.472 (2)
F2—C23	1.291 (3)	C9—C12	1.500 (2)
F3—C23	1.303 (4)	C10—H10A	0.9800
F4—C23	1.390 (3)	C10—H10B	0.9800
F2A—C23	1.632 (6)	C10—H10C	0.9800
F3A—C23	1.348 (13)	C11—H11A	0.9800
F4A—C23	1.223 (6)	C11—H11B	0.9800
O1—C5	1.233 (2)	C11—H11C	0.9800
O2—C13	1.215 (2)	C12—H12A	0.9800
O3—C13	1.3470 (19)	C12—H12B	0.9800
O3—C14	1.4607 (19)	C12—H12C	0.9800
N1—C1	1.370 (2)	C14—C15	1.508 (3)
N1—C9	1.394 (2)	C14—C16	1.511 (3)
N1—H1N	0.88 (2)	C14—H14A	1.0000
C1—C6	1.355 (2)	C15—H15A	0.9800
C1—C2	1.498 (2)	C15—H15B	0.9800
C2—C3	1.523 (2)	C15—H15C	0.9800
C2—H2A	0.9900	C16—H16A	0.9800
C2—H2B	0.9900	C16—H16B	0.9800
C3—C4	1.532 (2)	C16—H16C	0.9800
C3—H3A	0.9900	C17—C18	1.385 (2)
C3—H3B	0.9900	C17—C22	1.396 (2)
C4—C11	1.528 (2)	C18—C19	1.385 (2)
C4—C5	1.531 (2)	C19—C20	1.381 (3)
C4—C10	1.538 (2)	C19—H19A	0.9500
C5—C6	1.459 (2)	C20—C21	1.383 (3)
C6—C7	1.521 (2)	C20—H20A	0.9500
C7—C8	1.526 (2)	C21—C22	1.389 (2)
C7—C17	1.527 (2)	C21—C23	1.489 (3)
C7—H7A	1.0000	C22—H22A	0.9500
C8—C9	1.350 (2)		
			
C13—O3—C14	116.69 (13)	C9—C12—H12B	109.5
C1—N1—C9	121.29 (14)	H12A—C12—H12B	109.5
C1—N1—H1N	120.3 (14)	C9—C12—H12C	109.5
C9—N1—H1N	117.5 (14)	H12A—C12—H12C	109.5
C6—C1—N1	120.53 (15)	H12B—C12—H12C	109.5
C6—C1—C2	123.60 (15)	O2—C13—O3	123.17 (15)
N1—C1—C2	115.80 (14)	O2—C13—C8	126.25 (15)
C1—C2—C3	111.35 (14)	O3—C13—C8	110.58 (13)
C1—C2—H2A	109.4	O3—C14—C15	105.20 (14)
C3—C2—H2A	109.4	O3—C14—C16	108.92 (14)
C1—C2—H2B	109.4	C15—C14—C16	112.55 (16)
C3—C2—H2B	109.4	O3—C14—H14A	110.0
H2A—C2—H2B	108.0	C15—C14—H14A	110.0
C2—C3—C4	113.03 (14)	C16—C14—H14A	110.0
C2—C3—H3A	109.0	C14—C15—H15A	109.5
C4—C3—H3A	109.0	C14—C15—H15B	109.5
C2—C3—H3B	109.0	H15A—C15—H15B	109.5
C4—C3—H3B	109.0	C14—C15—H15C	109.5
H3A—C3—H3B	107.8	H15A—C15—H15C	109.5
C11—C4—C5	110.31 (13)	H15B—C15—H15C	109.5
C11—C4—C3	109.16 (14)	C14—C16—H16A	109.5
C5—C4—C3	109.75 (13)	C14—C16—H16B	109.5
C11—C4—C10	109.38 (15)	H16A—C16—H16B	109.5
C5—C4—C10	106.97 (14)	C14—C16—H16C	109.5
C3—C4—C10	111.26 (14)	H16A—C16—H16C	109.5
O1—C5—C6	120.70 (14)	H16B—C16—H16C	109.5
O1—C5—C4	120.64 (14)	C18—C17—C22	116.21 (15)
C6—C5—C4	118.56 (14)	C18—C17—C7	123.34 (15)
C1—C6—C5	121.08 (14)	C22—C17—C7	120.43 (15)
C1—C6—C7	119.93 (14)	F1—C18—C17	119.05 (15)
C5—C6—C7	118.91 (14)	F1—C18—C19	117.23 (16)
C6—C7—C8	109.00 (13)	C17—C18—C19	123.71 (18)
C6—C7—C17	111.51 (13)	C20—C19—C18	118.87 (18)
C8—C7—C17	110.85 (13)	C20—C19—H19A	120.6
C6—C7—H7A	108.5	C18—C19—H19A	120.6
C8—C7—H7A	108.5	C19—C20—C21	119.17 (17)
C17—C7—H7A	108.5	C19—C20—H20A	120.4
C9—C8—C13	120.85 (15)	C21—C20—H20A	120.4
C9—C8—C7	120.83 (14)	C20—C21—C22	121.04 (18)
C13—C8—C7	118.31 (14)	C20—C21—C23	119.70 (17)
C8—C9—N1	119.18 (14)	C22—C21—C23	119.26 (18)
C8—C9—C12	127.09 (15)	C21—C22—C17	120.99 (17)
N1—C9—C12	113.69 (14)	C21—C22—H22A	119.5
C4—C10—H10A	109.5	C17—C22—H22A	119.5
C4—C10—H10B	109.5	F2—C23—F3	111.3 (3)
H10A—C10—H10B	109.5	F4A—C23—F3A	111.0 (8)
C4—C10—H10C	109.5	F2—C23—F4	105.48 (18)
H10A—C10—H10C	109.5	F3—C23—F4	105.1 (2)
H10B—C10—H10C	109.5	F4A—C23—C21	125.0 (3)
C4—C11—H11A	109.5	F2—C23—C21	111.93 (16)
C4—C11—H11B	109.5	F3—C23—C21	113.0 (3)
H11A—C11—H11B	109.5	F3A—C23—C21	117.5 (9)
C4—C11—H11C	109.5	F4—C23—C21	109.55 (19)
H11A—C11—H11C	109.5	F4A—C23—F2A	93.9 (5)
H11B—C11—H11C	109.5	F3A—C23—F2A	88.7 (8)
C9—C12—H12A	109.5	C21—C23—F2A	111.1 (2)
			
C9—N1—C1—C6	16.7 (2)	C14—O3—C13—C8	177.28 (13)
C9—N1—C1—C2	−160.28 (16)	C9—C8—C13—O2	−14.5 (3)
C6—C1—C2—C3	16.0 (3)	C7—C8—C13—O2	166.88 (17)
N1—C1—C2—C3	−167.16 (16)	C9—C8—C13—O3	166.01 (15)
C1—C2—C3—C4	−47.4 (2)	C7—C8—C13—O3	−12.6 (2)
C2—C3—C4—C11	174.92 (14)	C13—O3—C14—C15	161.43 (15)
C2—C3—C4—C5	53.91 (19)	C13—O3—C14—C16	−77.70 (18)
C2—C3—C4—C10	−64.28 (19)	C6—C7—C17—C18	119.37 (17)
C11—C4—C5—O1	33.3 (2)	C8—C7—C17—C18	−118.98 (17)
C3—C4—C5—O1	153.65 (15)	C6—C7—C17—C22	−62.54 (19)
C10—C4—C5—O1	−85.53 (19)	C8—C7—C17—C22	59.10 (19)
C11—C4—C5—C6	−150.30 (15)	C22—C17—C18—F1	179.63 (14)
C3—C4—C5—C6	−30.0 (2)	C7—C17—C18—F1	−2.2 (2)
C10—C4—C5—C6	90.83 (18)	C22—C17—C18—C19	−0.7 (2)
N1—C1—C6—C5	−168.50 (15)	C7—C17—C18—C19	177.43 (16)
C2—C1—C6—C5	8.2 (3)	F1—C18—C19—C20	−178.98 (15)
N1—C1—C6—C7	8.1 (2)	C17—C18—C19—C20	1.4 (3)
C2—C1—C6—C7	−175.12 (16)	C18—C19—C20—C21	−1.2 (3)
O1—C5—C6—C1	175.91 (16)	C19—C20—C21—C22	0.4 (3)
C4—C5—C6—C1	−0.4 (2)	C19—C20—C21—C23	179.90 (17)
O1—C5—C6—C7	−0.8 (2)	C20—C21—C22—C17	0.3 (3)
C4—C5—C6—C7	−177.12 (14)	C23—C21—C22—C17	−179.26 (15)
C1—C6—C7—C8	−28.8 (2)	C18—C17—C22—C21	−0.1 (2)
C5—C6—C7—C8	147.86 (14)	C7—C17—C22—C21	−178.31 (15)
C1—C6—C7—C17	93.87 (18)	C20—C21—C23—F4A	71.2 (7)
C5—C6—C7—C17	−89.42 (17)	C22—C21—C23—F4A	−109.2 (7)
C6—C7—C8—C9	29.1 (2)	C20—C21—C23—F2	137.6 (2)
C17—C7—C8—C9	−93.96 (18)	C22—C21—C23—F2	−42.9 (3)
C6—C7—C8—C13	−152.26 (14)	C20—C21—C23—F3	−95.8 (3)
C17—C7—C8—C13	84.64 (18)	C22—C21—C23—F3	83.7 (3)
C13—C8—C9—N1	172.97 (15)	C20—C21—C23—F3A	−77.8 (8)
C7—C8—C9—N1	−8.5 (2)	C22—C21—C23—F3A	101.8 (8)
C13—C8—C9—C12	−4.9 (3)	C20—C21—C23—F4	21.0 (2)
C7—C8—C9—C12	173.69 (15)	C22—C21—C23—F4	−159.50 (17)
C1—N1—C9—C8	−16.5 (2)	C20—C21—C23—F2A	−177.7 (4)
C1—N1—C9—C12	161.63 (15)	C22—C21—C23—F2A	1.8 (4)
C14—O3—C13—O2	−2.2 (2)		

### Hydrogen-bond geometry (Å, º)

*Cg*3 is the centroid of the C17–C22 ring.

**Table:**

*D*—H···*A*	*D*—H	H···*A*	*D*···*A*	*D*—H···*A*
N1—H1*N*···O1^i^	0.88 (2)	2.12 (2)	2.9798 (19)	165 (2)
C2—H2*A*···F3^ii^	0.99	2.59	3.187 (4)	119
C12—H12*B*···F1^i^	0.98	2.64	3.206 (2)	117
C12—H12*B*···O1^i^	0.98	2.65	3.505 (2)	145
C12—H12*C*···F3*A*^iii^	0.98	2.43	3.243 (15)	141
C16—H16*A*···O2	0.98	2.59	3.106 (2)	113
C16—H16*C*···F4*A*^iv^	0.98	2.43	3.409 (6)	174
C19—H19*A*···F2^v^	0.95	2.52	3.117 (3)	121
C10—H10*C*···*Cg*3^ii^	0.98	2.93	3.631 (2)	130
C14—H14*A*···*Cg*3^iv^	1.00	2.91	3.7707 (18)	145

Symmetry codes: (i) *x*−1, *y*, *z*; (ii) *x*, −*y*+3/2, *z*+1/2; (iii) *x*, *y*, *z*+1; (iv) −*x*+1, −*y*+1, −*z*+1; (v) *x*+1, *y*, *z*.
